# Supplementary material for: Effect of nitrogen fertilizer on seed yield and quality of Kengyilia melanthera (Triticeae, Poaceae)
Source: PeerJ. 2022 Sep 22;10:e14101. doi: 10.7717/peerj.14101 (PMC9509668; doi:10.7717/peerj.14101)
Supplement: Supplemental Information 1 — The influence of N fertilizer on seed yield components, seed yield and quality in both trial years. [file peerj-10-14101-s001.docx]

| **Soil depth**  **/cm** | **pH** | **Organic matte**  **/g·kg^-1^** | **Total nitrogen**  **/g·kg^-1^** | **Total phosphorus**  **/g·kg^-1^** | **Total potassium**  **/g·kg^-1^** | **Available nitrogen**  **/mg·kg^-1^** | **Available phosphorus/mg·kg^-1^** | | **Available potassium /mg·kg^-1^** |
| --- | --- | --- | --- | --- | --- | --- | --- | --- | --- |
| 0~30 | 6.02 | 13.8 | 1.32 | 0.86 | 0.68 | 87.47 | 62.55 | 227.46 | |
| 30~60 | 6.14 | 14.7 | 0.81 | 0.74 | 0.72 | 74.38 | 51.37 | 207.56 | |
