# Supplementary material for: Effect of nitrogen fertilizer on seed yield and quality of Kengyilia melanthera (Triticeae, Poaceae)
Source: PeerJ. 2022 Sep 22;10:e14101. doi: 10.7717/peerj.14101 (PMC9509668; doi:10.7717/peerj.14101)
Supplement: Supplemental Information 2 — Note. NTs, number of tillers; NFTs, number of fertile tillers; NSPs, number of spikes; NFLs, number of florets; NFFLs, number of fertile florets; TSW, 1,000-seed weight; PSY, potential seed yield; PRSY, presentation seed yield; HSY, harvested seed yield; SGR, standard germination rate; GE, germination energy; AAGR, accelerated aging germination rate; DE, dehydrogenase; APH, acid phosphoesterase; N1 – N fertilizer 0 kg·hm−2 (Control); N2 – N fertilizer 60 kg·hm−2; N3 – N fertilizer 90 kg·hm−2; N4 – N fertilizer 120 kg·hm−2; N5 – N fertilizer 150 kg·hm−2; N6 – N fertilizer 180 kg·hm−2; N7 – N fertilizer 210 kg·hm−2; N8 – N fertilizer 240 kg·hm−2. [file peerj-10-14101-s002.docx]

| **Parameter** | **N fertilizer treatments** | | | | | | | |
| --- | --- | --- | --- | --- | --- | --- | --- | --- |
|  | **N_1_** | **N_2_** | **N_3_** | **N_4_** | **N_5_** | **N_6_** | **N_7_** | **N_8_** |
| NTs/m^2^ | 0.00 | 0.06 | 0.41 | 0.61 | 0.65 | 1.00 | 0.97 | 0.91 |
| NFTs/m^2^ | 0.00 | 0.03 | 0.48 | 0.61 | 0.76 | 0.90 | 1.00 | 0.98 |
| TSW/g | 0.00 | 0.33 | 0.38 | 0.62 | 0.91 | 1.00 | 0.96 | 0.90 |
| NSPs per fertile tillers | 0.00 | 0.00 | 0.05 | 0.55 | 0.75 | 1.00 | 0.70 | 0.50 |
| NFLs per spike | 0.00 | 0.27 | 0.47 | 0.67 | 0.93 | 1.00 | 0.87 | 0.40 |
| NFFLs per spike | 0.00 | 0.00 | 0.20 | 0.40 | 0.80 | 1.00 | 0.60 | 0.20 |
| PSY | 0.00 | 0.08 | 0.38 | 0.59 | 0.82 | 1.00 | 0.96 | 0.78 |
| SSY | 0.00 | 0.06 | 0.34 | 0.57 | 0.79 | 1.00 | 0.94 | 0.80 |
| ASY/kg·hm^-2^ | 0.00 | 0.18 | 0.23 | 0.43 | 0.67 | 1.00 | 0.89 | 0.73 |
| SGR/% | 0.00 | 0.50 | 1.00 | 0.50 | 1.00 | 0.50 | 1.00 | 0.50 |
| GE/% | 0.50 | 0.75 | 0.00 | 0.25 | 0.25 | 1.00 | 0.75 | 0.75 |
| AAGR/% | 0.50 | 0.50 | 1.00 | 0.50 | 0.25 | 0.50 | 1.00 | 0.00 |
| DE activity/µg·mL^-1^ | 0.00 | 0.21 | 0.47 | 0.88 | 0.62 | 1.00 | 0.96 | 0.55 |
| APH activity/nmol·min^-1^. 50 seeds | 0.20 | 0.00 | 0.90 | 0.50 | 1.00 | 0.80 | 0.90 | 0.70 |
| Rank | 8 | 7 | 6 | 5 | 3 | 1 | 2 | 4 |
